# Characterization of influenza seasonality in China, 2010–2018: Implications for seasonal influenza vaccination timing

**DOI:** 10.1111/irv.13047

**Published:** 2022-09-05

**Authors:** Yilan Liao, Shan Xue, Yiran Xie, Yanping Zhang, Dayan Wang, Tong Zhao, Wei Du, Tao Chen, Hui Miao, Ying Qin, Jiandong Zheng, Xiaokun Yang, Zhibin Peng, Jianxing Yu

**Affiliations:** ^1^ State Key Laboratory of Resources and Environmental Information System, Institute of Geographic Sciences and Natural Resources Research Chinese Academy of Sciences Beijing China; ^2^ College of Resources and Environment University of Chinese Academy of Sciences Beijing China; ^3^ Chinese National Influenza Center National Institute for Viral Disease Control and Prevention, Chinese Center for Disease Control and Prevention Beijing China; ^4^ Division of Infectious Diseases Chinese Center for Disease Control and Prevention Beijing China; ^5^ School of Public Health Southeast University Nanjing China; ^6^ College of Art and Science Ohio State University Columbus Ohio USA; ^7^ National Institute for Communicable Disease Control and Prevention Chinese Center for Disease Control and Prevention Beijing China

**Keywords:** China, influenza, seasonality, spatial heterogeneity, vaccination

## Abstract

**Background:**

Optimizing the timing of influenza vaccination based on regional temporal seasonal influenza illness patterns may make seasonal influenza vaccination more effective in China.

**Methods:**

We obtained provincial weekly influenza surveillance data for 30 of 31 provinces in mainland China from the Chinese Center for Disease Control and Prevention for the years 2010–2018. Influenza epidemiological regions were constructed by clustering analysis. For each region, we calculated onset date, end date, and duration of seasonal influenza epidemics by the modified mean threshold method. To help identify initial vaccination target populations, we acquired weekly influenza surveillance data for four age groups (0–4, 5–18, 19–59, and ≥60 years) in each region and in 171 cities of wide‐ranging size. We used linear regression analyses to explore the association of epidemic onset dates by age group, city, and epidemiological region and provide evidence for initial target populations for seasonal influenza vaccination.

**Results:**

We determined that northern, mid, southwestern, southeast regions of mainland China have distinct seasonal influenza epidemic patterns. We found significant regional, temporal, and spatial heterogeneity of seasonal influenza epidemics. There were significant differences by age group and city size in the interval between epidemic onset in the city or age group and regional spread (epidemic lead time), with longer epidemic lead times for 5‐ to 18‐year‐old children and larger cities.

**Conclusions:**

Knowledge of influenza epidemic characteristics may help optimize local influenza vaccination timing and identify initial target groups for seasonal influenza vaccination in mainland China. Similar analyses may help inform seasonal influenza vaccination strategies in other regions and countries.

## 1 INTRODUCTION

Influenza is a consistently important threat to global health. Every year, an estimated 1 billion influenza‐related respiratory illnesses, 3–5 million severe cases, and 290,000–650,000 deaths are caused by seasonal influenza worldwide.^1^ In the mainland of China, influenza caused an average of 88,000 excess respiratory deaths each year during the 2010–2011 through 2014–2015 influenza seasons.^2^

Influenza vaccination is the most effective intervention to prevent influenza disease. However, protective effectiveness varies from year to year, likely influenced by many factors such as timing of vaccination, age of target populations, match between vaccine and circulating strains, thermal inactivation, and egg adaptations in vaccine chicken embryo strains.^3^ Two phenomena that decrease protective antibody levels and lessen duration of protective effectiveness are influenza virus' rapid genetic mutation rate, leading to escape from existing immunity, and rapid waning of influenza vaccine‐induced immunity.^4^ Therefore, it is prudent to time comprehensive influenza vaccination effort with considerations of season, local conditions, and target populations to optimize protective impact of influenza vaccines.

Studies conducted in China have found differences in influenza seasonality in temperate, subtropical, and tropical regions.^5^ However, core parameters for predicting influenza seasonality in different regions of China, which are important for local government formulation of control and prevention measures and timing of influenza vaccination, have been insufficiently studied. After the novel influenza A/H1N1 virus (H1N1pdm09) emerged in 2009, it supplanted previous human H1N1 viruses to become a seasonal human H1N1 virus.^6^ It is unknown how circulation of novel influenza viruses affects influenza seasonality. Therefore, to improve the current vaccination strategy, which was formulated with national 2005–2011 influenza seasonality in mind, it is useful for public health policy makers to understand important changes in influenza seasonality during the most recent decade.

We used influenza surveillance data from 30 of mainland China's 31 provinces during 2010 to 2018 to explore variation in seasonal influenza epidemics and estimate epidemic characteristic parameters by epidemiological region, age group, and city size to provide evidence for optimizing seasonal influenza vaccination timing, target populations, and locations We believe that findings may provide new ideas for improving the current vaccination timing in China and other countries.

## 2 METHODS

### 2.1 Data and epidemiological region

Since 2009, the Influenza Surveillance Network has covered all prefecture‐level cities in mainland China, including 556 sentinel hospitals,^7^ which collected epidemiologic and laboratory surveillance data on outpatient and emergency influenza‐like illness (ILI) patients. The ILI case identification conforms to a standard definition: temperature ≥38°C with either cough or sore throat.^6^ Respiratory specimens were collected from a subset of ILI patients and transported to influenza surveillance network laboratory within 48 h for influenza virus testing. The influenza network laboratories conducted testing by real‐time Reverse Transcription‐ Polymerase Chain Reaction (rRT‐PCR) following the standard protocol.^8^, ^9^ Table 1 shows influenza sampling intensity by province.

**TABLE 1:** Characteristics of the 30 study provinces and their sampling intensities, 2010–2018

	Influenza epidemiological region	Centroid latitude (°N)	Centroid longitude (°E)	Population size (million)	Average weekly counts of tested specimens	Average weekly number of influenza positive specimens by rRT‐PCR
Heilongjiang	Northern	47.9	127.8	38.3	229.2	23.7
Inner Mongolia	Northern	44.1	113.9	24.7	164.4	19.1
Jilin	Northern	43.7	126.2	27.5	137.0	16.7
Liaoning	Northern	41.3	122.6	43.7	234.1	20.8
Xinjiang	Northern	41.1	85.2	21.8	202.2	24.4
Beijing	Northern	40.2	116.4	19.6	203.5	32.4
Hebei	Northern	39.6	116.1	71.9	260.0	36.9
Tianjin	Northern	39.3	117.3	12.9	100.4	20.2
Gansu	Northern	37.9	100.5	25.6	200.6	30.4
Shanxi	Northern	37.6	112.3	35.7	169.7	28.6
Ningxia	Northern	37.3	106.2	6.3	97.0	12.3
Shandong	Northern	36.4	118.1	95.8	306.7	36.4
Qinghai	Northern	35.7	96.0	5.6	92.9	9.6
Shaanxi	Northern	35.2	108.9	37.3	199.8	30.5
Henan	Northern	33.9	113.6	94.0	237.8	38.5
Jiangsu	Mid	33.0	119.4	78.7	507.7	58.9
Anhui	Mid	31.8	117.2	59.5	372.3	53.5
Shanghai	Mid	31.1	121.4	23.0	321.2	79.0
Hubei	Mid	31.0	112.2	57.2	294.2	39.3
Zhejiang	Mid	30.6	102.7	54.4	264.3	52.6
Hunan	Mid	30.0	107.9	65.7	375.0	38.1
Chongqing	Southwestern	29.2	120.0	28.8	106.7	15.8
Sichuan	Southwestern	27.6	115.7	80.4	351.4	46.8
Guizhou	Southwestern	27.6	111.7	34.7	222.1	28.6
Yunnan	Southwestern	26.8	106.9	46.0	314.1	25.7
Jiangxi	Southeastern	26.1	118.0	44.6	247.6	37.7
Fujian	Southeastern	25.0	101.5	36.9	232.1	37.0
Guangdong	Southeastern	23.8	108.8	104.3	493.7	61.4
Guangxi	Southeastern	23.4	113.4	46.0	275.8	34.5
Hainan	Southeastern	19.2	109.7	8.7	95.8	9.8

We summarized the number of weekly province‐level ILI samples tested for influenza and influenza virus positive isolates between September 27, 2010 and May 27, 2018, after the end of 2009 H1N1 influenza pandemic. We defined week 40 to week 39 the following year as an influenza season. The 2017–2018 influenza season only included 46 weeks; no surveillance data were available for Hong Kong, Macao, and Taiwan; Tibet was excluded due to insufficient number of samples, as fewer than 10 specimens were tested in Tibet in 48% of the reporting weeks.

To adjust for differences in the number of ILI samples tested for influenza, we standardized province‐level weekly counts of positive isolates by the annual number of influenza specimens tested using Equation (1).

(1) $$P_{n,s,i}=\frac{O_{n,s,i}}{\sum_{i\in I}T_{n,s,i}}$$

where I is the set of weeks in an influenza season and $P_{n,s,i}$, $O_{n,s,i}$, and $T_{n,s,i}$ are weekly standardized counts of influenza virus positive isolates, the number of influenza virus positive isolates, and the number of influenza specimens tested in province n in the $i^{\mathrm{th}}$ week of influenza season s, respectively. To show temporal and spatial differences visually, we calculated the average weekly standardized counts of influenza positive isolates to indicate the relative intensity of influenza activity and made relative intensity into a heat map.

To help design an accurate vaccination program by providing local condition evidence, based on average weekly standardized counts of influenza positive isolates from each province, we categorized influenza epidemiological regions in China using hierarchical clustering with cosine distance as a clustering statistic and Ward's minimum variance as an intercluster distance.

### 2.2 Epidemic parameters by epidemiological region

To characterize influenza epidemics and stable influenza activity in pre‐specified influenza epidemiological regions, we summed weekly standardized counts of influenza virus positive isolates in provinces belonging to the same region weighted by population to obtain weekly counts of influenza virus positive isolates for the region.

We used a modification of Azziz Baumgartner and colleagues^10^ method to define the onset, end, and the number of epidemics in each season of the four influenza epidemiological regions. If the standardized positive count was higher than the average threshold for 3 weeks, and the third week's standardized positive count was greater than the first week's standardized positive count, the first week was considered to be the onset week of an epidemic period. Similarly, if there were three consecutive weeks below average level after the epidemic onset, the first week was defined as the end of the epidemic period.

And then influenza seasonality patterns during the eight seasons for each epidemiological region were determined. A week was defined as having stable influenza activity if it was determined as epidemic week in at least three of the eight study seasons, the first and last weeks of continuous influenza activity were defined as the onset and end of influenza activity.^7^ Seasonality characteristics in the influenza epidemiological regions were then classified into three types: (a) having one peak, characterized as having 1–7 months of continuous influenza activity once a year; (b) having two peaks, characterized as two sets of influenza activity each lasting at least 1 month long and having at least a 2‐month gap without influenza activity in between; and (c) having year‐round activity, characterized as 8+ months of influenza activity or 3+ influenza activity lasting at least 1 month long and having at least a 2‐month gap without influenza activity in between.

### 2.3 Regional lead times by city size and age group

To help identify initial vaccination target populations, we calculated the number of weeks the annual epidemic precedes regional spread by age group and city size for each epidemiological region. We call this value the “lead time.” Shown as Table 2, we categorized age into four groups (0–4, 5–18, 19–59, and 60 + years). The group 5–18 instead of 5–17 was chosen because several studies have found that winter or summer vacation has a significant impact on the number of influenza‐positive cases, especially among children and adolescents.^11^, ^12^, ^13^ And some high school third‐year students in China happened to be 18 years old. The number of samples tested and number of positive isolates for each age group in each region were determined by summing corresponding age group values in the region.

**TABLE 2:** Age groupings and their testing and positivity frequencies

	0–4	5–18	19–59	≥60
Average weekly counts of tested specimens	737.5	435.8	526.4	103.7
Average weekly number of influenza positive specimens by rRT‐PCR	72.8	79.3	77.9	15.8

Abbreviation: rRT‐PCR, real‐time Reverse Transcription‐ Polymerase Chain Reaction.

To determine whether there were earlier influenza epidemics in certain types of cities, we first categorized cities in the 30 provinces into five types based on their population size, in accordance with the Notice on Adjusting the Criteria for City Sizes Classification published by China State Council.^8^ Cities missing≥5% of influenza surveillance data were excluded from the study; 171 cities remained in our dataset. Table 3 shows city size criteria of population size and the numbers of each type of city.

**TABLE 3:** City size criteria and distribution

	Megacity	Supercity	Type I large city	Type II large city	Medium‐and small‐sized city
Resident population in urban areas	≥10 million	5–10 million	3–5 million	1–3 million	≤1 million
Number of cities	6	9	10	39	107
Average weekly counts of tested specimens	137.7	44.0	44.4	28.9	19.5
Average weekly number of influenza positive specimens by rRT‐PCR	26.6	6.3	5.7	3.8	2.5

Abbreviation: rRT‐PCR, real‐time Reverse Transcription‐ Polymerase Chain Reaction.

For each region, we determined the regional epidemic onset and the specific lead times for each of the four age groups and each of the study cities in the respective regions. To test whether there were statistically significant differences in lead times by city size and age group, we used non‐parametric tests for between and within group differences. The non‐parametric Kruskal–Wallis tests followed by Dunn's multiple‐comparison tests were applied for multiple comparisons. We used correlation tests to determine whether lead times were related to urban resident population size for the 177 cities.

## 3 RESULTS

### 3.1 Temporal and spatial heterogeneity by province

**FIGURE 1:**
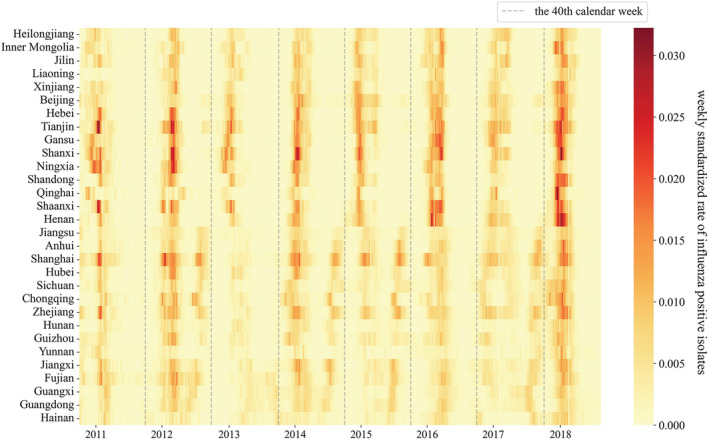
Spatiotemporal heat map of weekly standardized counts of influenza positive isolates from 2010 to 2018. Provinces are arranged from north to south by their central latitudes. The gray dotted lines denote the 40th calendar weeks (the start of epidemic seasons).

**FIGURE 2:**
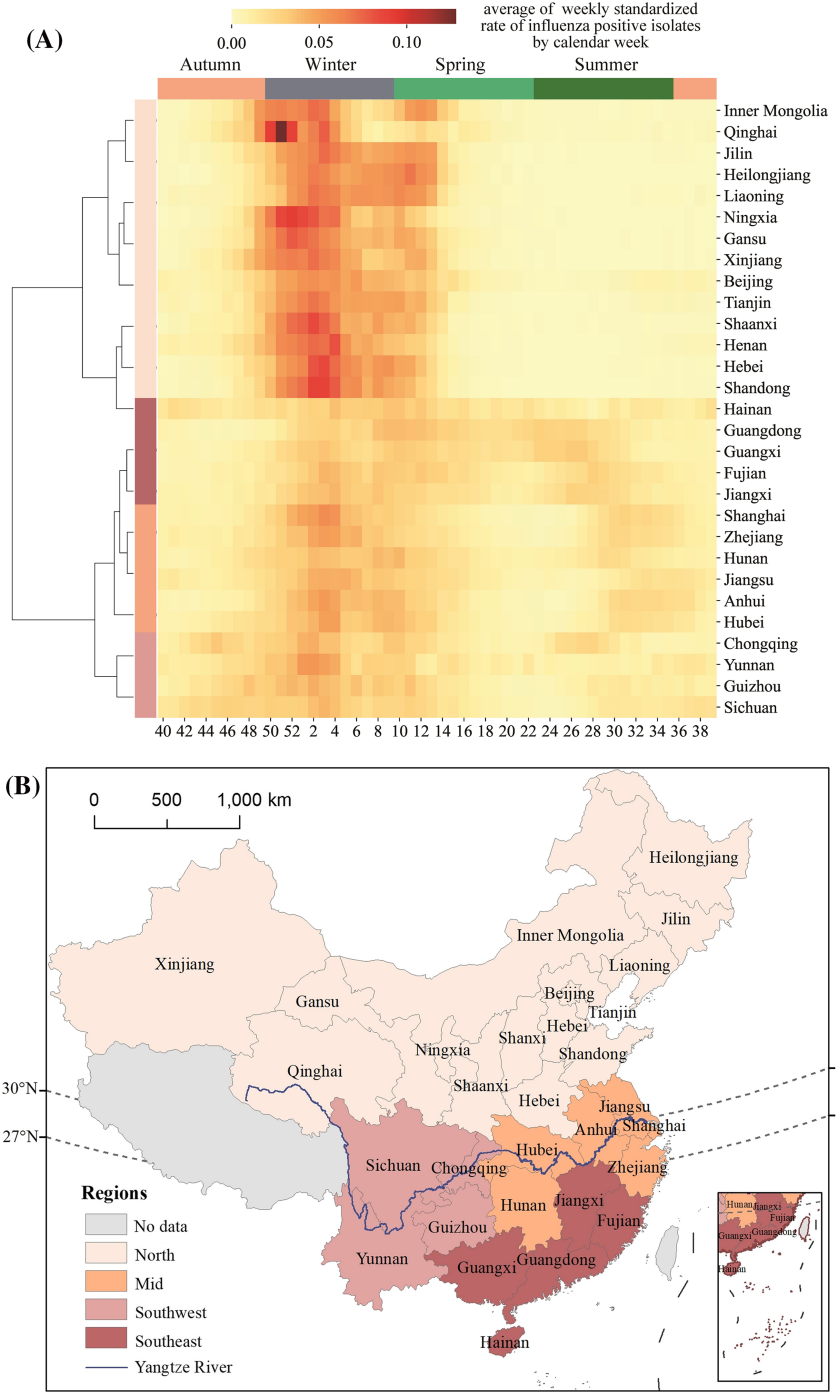
Influenza epidemiological regions and spatiotemporal heat map of average weekly standardized counts of influenza positive isolates. (A) Hierarchical clustering and spatiotemporal heat map of average weekly standardized counts of influenza positive isolates for each province. (B) Map of the four epidemiological regions defined in (A)

Figure 1 is a spatiotemporal heat map of the weekly standardized counts of influenza positive isolates from 2010 to 2018, and Figure 2A is a heat map of average weekly standardized counts of influenza positive isolates in one epidemic season. It is shown that northern provinces, typically north of Henan province (33°N), experience high weekly standardized positive counts in winter and spring and have influenza outbreaks that are more clustered than in southern provinces without significant changes in the entire period. In contrast, Jiangsu province and the southern provinces, typically south of 33°N, had stable high weekly standardized positive counts in winter, spring, and intermittent high weekly standardized positive counts in summer. The similar phenomenon could also be observed in the southernmost provinces (south of 27°N), while the gap between peak weekly standardized positive counts in winter, spring, and summer was not apparent in these provinces. The heat map shows long‐lasting periods of activity in winter to summer in the southern provinces, which differs from previous findings of a single peak appearing in April to June in the southernmost provinces.^5^

Figure 2B shows the hierarchical clustering pattern of “north‐mid‐southwest‐southeast” for influenza virus activity. The northern region includes provinces to the north of 33°N in the temperate zone. The southwestern region includes provinces at 27.4°N to 33°N, 93°E to 110°E, including Sichuan and Chongqing in the upper reaches of the Yangtze River and Yunnan and Guizhou in the southwest of China. The mid region includes provinces at 27.4°N to 33°N, 110°E to 123°E, including provinces located in the middle and lower reaches of the Yangtze River. The southeastern region includes provinces to the south of 27°N.

### 3.2 Epidemic parameters by region

**FIGURE 3:**
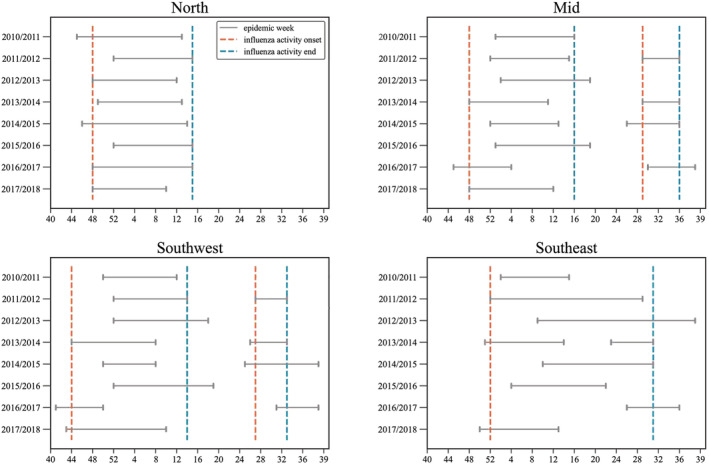
Epidemic weeks and onset and end weeks in each region. Gray solid lines denote epidemic weeks for each influenza season. Brown and blue dotted lines represent onset and end of influenza activities for each region.

Figure 3 and Table 4 show that from 2010 to 2018, peak influenza activity occurred once a year in the northern region, indicating that influenza appears to be one‐peak epidemic in northern China. Influenza activity most commonly occurred in the winter–spring seasons during the 48th week in a current year to the 15th week the following year in the northern region, thus covering 20 weeks of a typical influenza season each year. There was a mixed pattern in the mid and southwestern regions, where one‐peak epidemics appeared in half of the influenza seasons and two‐peak epidemics occurred in the other seasons. Mixed one‐peak and two‐peak patterns were similar in the mid and southwestern regions. The peak time of winter influenza virus activity in the southwestern region was during the 44th week in a current year to the 14th week of the following year, while the winter influenza activity peaked during the 48th week to the 16th week the following year in the mid region. Summer influenza virus activity peaked during the 27th to the 33rd week in the southwestern region, while it peaked during the 29th to the 36th week in the mid region. Thus, the mid region epidemic peak lagged the southwestern region epidemic peak.

**TABLE 4:** Epidemic pattern and onset and end timing of influenza epidemic activity in each region

	Northern region	Mid region		Southeastern region	Southwestern region	
Epidemic pattern	One peak	Two peaks		Year‐round	Two peaks	
Starting time of epidemic activity (calendar week)	48	48	29	52	44	27
Ending time of epidemic activity (calendar week)	15	16	36	31	14	33
Epidemic duration (weeks)	20	21	8	32	23	7

**FIGURE A1:**
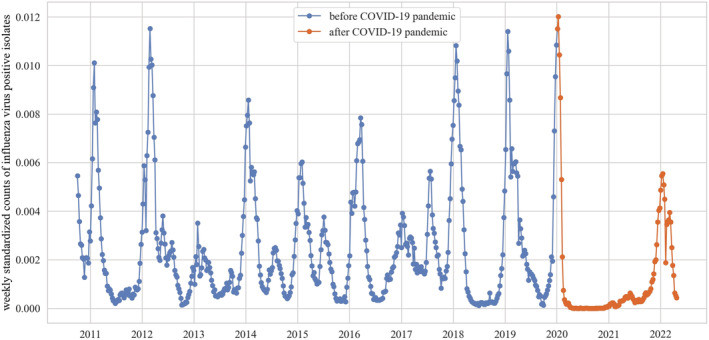
Weekly standardized counts of influenza virus positive isolates in China from 2010/2011 influenza season to the 16th week of 2022. The counts of influenza virus positive isolates and the counts of influenza specimens tested were obtained from the World Health Organization (WHO, https://apps.who.int/flumart/Default?ReportNo=12) and calculated the weekly standardized counts of influenza virus positive isolates according to the method in this study.

Seasonality of influenza in the southeastern region was more complex than seasonality in the other three regions, especially considering that the distribution of the southeastern epidemic period varied by epidemic season. While the epidemic season in the southeastern region was similar to the two‐peak, winter‐and‐summer patterns in the southwestern and mid regions in 2013–2014, it appeared to be an annual one‐peak pattern in the other seven epidemic seasons. Seasonality patterns in 2010/2011 and 2017/2018 in the southeastern region were similar to the winter epidemic activity during December to March in the northern region. The pattern during 2014/2015 and 2015/2016 seasons in the southeastern region differed from the northern region, with the epidemic periods concentrated in March through May. There was no winter epidemic peak in the 2013/2014 season, and the epidemic peaked in summer from June to August. During the 2011/2012 and 2012/2013 epidemic seasons, the epidemic lasted for more than half a year (29 weeks and 30 weeks, respectively) and spread over three seasons (winter, spring, and summer and spring, summer, and autumn; respectively). The influenza outbreaks in the southeastern region showed some randomness, and timing and duration of the epidemic periods were therefore less predictable. Influenza virus activity peaked from the 52nd to the 31st week over winter, spring, and summer, demonstrating an all‐year epidemic pattern. We also calculated epidemic time and for the whole of China before and after the COVID‐19 pandemic (see in Table A1 and Figure A1), indicating that seasonal influenza activity in China is gradually returning to the seasonal pattern before the COVID‐19 pandemic.

### 3.3 Epidemic lead times

**FIGURE 4:**
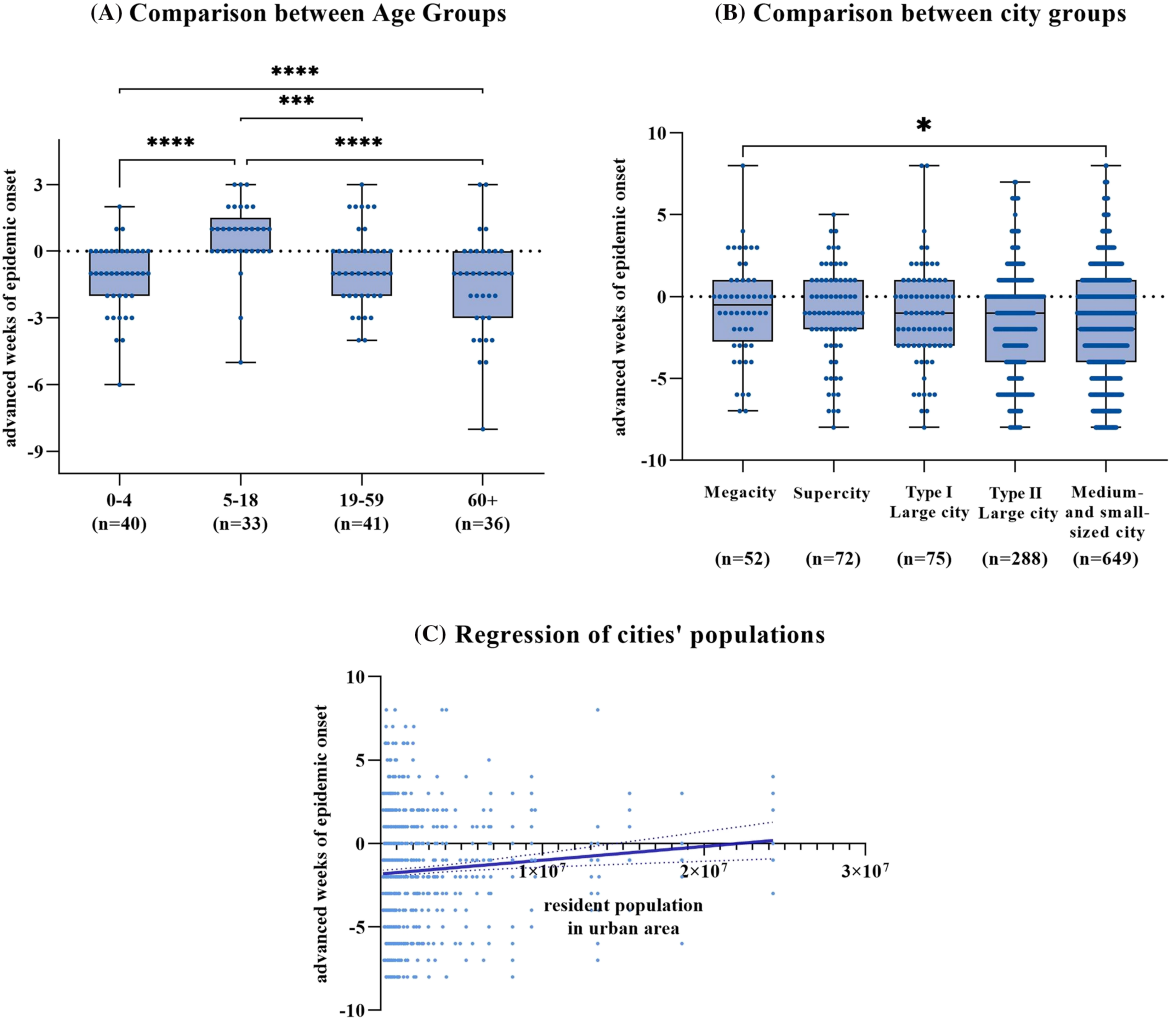
Lead times by age group and city size. (A) Boxplot of lead time for the four age groups. Betweengroup testing by Kruskal–Wallis test and multiple comparisons by Dunn's test. *, ***, and **** denote significant levels of adjusted *p* value<0.05, *p* value <0.0005, and *p* value <0.0001, respectively. (B) Boxplot of lead time for different size cities. (C) Regression plot of advanced epidemic onset and resident population in urban areas

There was variation in lead time by age group (Figure 4), with epidemics starting earliest among school‐age children, 5 to 18 years old, than among other age groups—an average of 1.3 weeks earlier (95% CI = 0.56–2.1 weeks).

Epidemic starting times varied by city population size; non‐parametric tests showed significant difference in lead time by city size. City population size was positively correlated with lead time (Pearson's *r* = 0.11, *p* < 0.01), indicating that the annual epidemics start earlier in larger cities than those in smaller cities.

## 4 DISCUSSION

Our study of the epidemiology of seasonal influenza in mainland China found that from 2010 to 2018, the northern China provinces had annual single‐peak seasons during winter and spring; the mid and southwest provinces had two peak seasons in winter and summer, with the southwest provinces preceding the mid region provinces by 3 to 4 weeks, and the southeastern provinces had little seasonality with a quasi‐random pattern of outbreaks, including one‐peak, two‐peak, and year‐round epidemics. Our study further showed that annual epidemics tend to start with school‐age children leading the epidemic onsets of other age groups, ahead of regional spread. Similarly, larger cities epidemic onsets lead smaller city onsets, with epidemic lead time positively correlated with city size, although the epidemic onset for the majority of cities would fall behind the average lead time. Findings from our study can be used to improve timing of vaccination target groups to help optimize protection of the population from influenza and its complications.

Our study estimated separately the influenza seasonal characteristic parameters in different epidemiological regions during the study period. We then identified suitable influenza vaccination timing and target populations for each region. We identified four influenza epidemiological regions—north, mid, southwest, and southeast—based on province‐level average weekly standardized counts of influenza positive isolates during the eight‐season study period. The original mid epidemiological region identified by previous study^5^ was divided into the southwestern reggion at 27.4°N to 33°N, 93°E to 110°E and the mid region at 27.4°N to 33°N, 110°E to 123°E. Our results confirmed the existence of seasonality differences between these two epidemiological regions. The regions in our study were based on province‐level, which would be helpful to provide more pertinence and specificity guidance for vaccination. Other studies on influenza seasonality in the mid region have found differences of seasonality between southwestern provinces and other provinces in the mid epidemiological region.^9^, ^14^

Influenza seasonal characteristic parameters can provide background information to formulate and implement the national vaccination strategy and other prevention and control measures, such as clinical investigations and public health surveillance.^11^, ^15^ We calculated the onset, end, and duration of influenza epidemics during the period of 2011 to 2018 in each epidemiological region. Our finding of a one‐peak winter–spring epidemic in northern China is consistent with studies from other countries in similar temperate regions, including the United States, the United Kingdom, and European countries.^12^ Although the onset of influenza epidemics in the mid region occurred 3–4 weeks later than in the southwestern region, the influenza epidemic seasons in those two regions shared characteristics of two‐peak, winter‐and‐summer patterns. Similar patterns were observed in the Philippines, Thailand, India, Pakistan, Hong Kong, Australia, and other countries and regions similar in latitude to China's subtropical regions.^13^, ^15^ Influenza epidemic patterns in the southeastern region of China, with little seasonality and one‐peak, two‐peak, and year‐round activity epidemic patterns are similar to seasonality found in the regions near the equator, including parts of Southeast Asia, Africa, and South America.

Compared with influenza seasonal parameters calculated in previous studies,^5^, ^16^ we found longer influenza activity in winter and spring in northern, mid, and southwestern regions (December–March in this study but January–February in Yu et al.^5^) and shorter duration influenza activity in summer and autumn in mid and southwestern regions (July–August in this study but June–August in Yu et al.^5^). The greatest difference appeared in the epidemic parameters of the southeastern region. In contrast to the two distinct patterns proposed by Yu et al.^5^ (one peak in April–June) and Zou et al.^16^ (high summer peaks and slightly lower winter peaks), we found a more diverse distribution of epidemics between the eight influenza seasons, in that influenza activity spanned winter, spring, and summer seasons. A recent systematic review and spatio‐temporal meta‐analysis on seasonality of influenza in China also point out the semi‐annual epidemics or year‐round activity in mid‐lattitude and low‐lattitude provinces.^16^ In contrast, our study used more detailed and complete weekly influenza surveillance data rather than data extracted from figures and tables. Influenza epidemiological regions and influenza activity time, seasonal pattern for each region, were provided in detail, which is of great significance to make national implementation strategies in China.

The current guidelines issued by China CDC, Technical guidelines for seasonal influenza vaccination in CDC (2020–2021), recommend completion of the influenza vaccination before the end of October. However, the public health department should offer vaccination service to people who have not been vaccinated throughout the entire influenza season.^17^ The recommend vaccination schedule covers at least one epidemic peak time in all epidemiological regions and can protect people effectively during influenza epidemic peaks occurring in winter and spring. However, because of the summer influenza epidemic peak south of 33°N, influenza vaccines may fail to provide sufficient protection against the summer epidemic if people there are vaccinated before the previous winter. Hence, public health departments should pay attention to the summer epidemic peak caused by influenza and adopt evidence‐based intervention strategies, such as strengthening surveillance and preventing nosocomial transmission. Clinical trials in Hong Kong, with two‐peak influenza epidemics each year, and a cost‐effectiveness model of vaccination in Singapore, which has little seasonality, have evaluated biannual influenza vaccination. These studies found that biannual vaccination provides better protection in the summer with superior cost‐effectiveness for the elderly.^7^, ^18^, ^19^ Shortening the influenza vaccination cycle is recommended to improve immunity and reduce harm, especially among the elderly and other vulnerable populations. However, such a strategy will pose a great challenge for influenza vaccine manufacturers because the current production cycle of influenza vaccine takes too long to produce a vaccine within half a year. Influenza activities in southeast China do not appear stable, which calls for influenza vaccination in this region throughout the year. The public health department should strengthen influenza surveillance to capture information on circulating influenza strains to improve the antigenic match between the influenza vaccine strains and the circulating virus strains.

Precise identification of target populations is another important factor that may influence the effectiveness of seasonal influenza vaccination. The current seasonal influenza vaccination strategy in China defines priority groups for vaccination from occupational and exposure perspectives. Occupational and exposure perspectives focus on local surroundings and micro‐environments. However, our study found differences by city size in lead time between city epidemic onset and regional spread. Previous work in the United States and Australia suggests high geographical correlation of influenza epidemics at a local level and that populous cities tend to have well‐synchronized influenza epidemics.^20^, ^21^ Studies on the epidemiology and activity of influenza viruses in Madrid and Algeria showed there were higher proportions of specimens testing positive for influenza in school‐age children compared with other age groups.^22^, ^23^ Therefore, ILI monitoring targeting school children (aged 5–18 years) and populous cities has potential to detect changes in influenza activity intensity earlier and faster. In addition to helping to focus surveillance, these findings provide evidence supporting adjustment and optimization of vaccination target population timing and optimization of timing of other public health interventions.

Strengths of our study include that we used a very large dataset with a long monitoring time, and wider coverage, thus providing the ability to determine seasonal parameters not determined previously in China, such as city‐level and age‐level lead times. Consistent with a study conducted by Yu and colleagues in 2013,^5^ we observed variation of influenza seasonality in different regions of China. Our study quantified the seasonal patterns, onset, and end of influenza activities and changes of influenza epidemics during 2010 to 2018 in China. Considering seasonal instability of influenza in China's subtropical and tropical region to the south of 33°N, our study used methods based on epidemic thresholds, which have been widely used for retrospective and real‐time influenza time series analyses.^10^, ^24^, ^25^ We believe, therefore, that our study has practical significance for formulation of national and regional public health policies and optimization of vaccination and other influenza prevention and control strategies.

In the subtropical and tropical regions (latitude lower than 33°N), seasonality of influenza is relatively unstable. Wavelet analysis showed that in addition to the northern region, the remaining three regions' seasonality of influenza periodicity was not stable and poses a challenge for static linear regression methods. However, analyses based on epidemic thresholds can accurately capture the characteristics of influenza epidemic periods for a single influenza season, better reflect variation of seasonality, and summarize regional influenza epidemic patterns and seasonal characteristic parameters.

Our study has several limitations. First, the quantity of data from Tibet was insufficient, precluding inclusion in our study. Second, we considered standardized positive counts to reflect the intensity of influenza activity, which can be affected by the denominator, potentially leading to distortion of influenza seasonal characteristic parameters. Third, considering national implementation strategies, we simplified complex measures and did not analyze the seasonality of influenza at the provincial and sentinel hospital levels. The influenza seasonal characteristic parameters obtained in the study may not be optimal at the provincial or hospital level but should be useful at the regional level. Our study did not explore factors driving observed seasonality. Possible explanations are worthy of close attention and deserve further investigation to reveal differences in seasonal characteristics between different influenza subtypes.

## 5 CONCLUSIONS

In conclusion, we determined seasonal influenza characteristics parameters in China. These parameters can be of value to optimize influenza vaccine vaccination policies and formulate targeted influenza detection and public health intervention strategies. The vaccination and other influenza prevention and control strategies should be optimized according to locally specific parameters. Moreover, public health departments should pay attention to seasonal variation of influenza characteristics parameters for school children (aged 5–18 years) and populous cities. Vaccination timing of target population should be adjusted and optimized timely. With gradual improvement of influenza vaccine coverage in China, these parameters can be considered by policy makers for effectively reducing the burden of influenza and reducing harm of influenza to the health of China's people .

## AUTHOR CONTRIBUTIONS

**Yilan Liao:** Conceptualization; methodology. **Shan Xue:** Data curation; formal analysis. **Dayan Wang:** Validation. **Tong Zhao:** Data curation; formal analysis. **Wei Du:** Validation. **Tao Chen:** Data curation; formal analysis. **Zhibin Peng:** Conceptualization. **Jianxing Yu:** Conceptualization.

## CONFLICT OF INTEREST

The authors declare that they have no competing interests.

### PEER REVIEW

The peer review history for this article is available at https://publons.com/publon/10.1111/irv.13047.

## Data Availability

The weekly standardized counts of influenza positive isolates in four regions analyzed in this study are publicly available online (https://zenodo.org/record/6964010#.YuwAhmNByUk).

## APPENDIX A

**TABLE A1:** Epidemic pattern and onset and end timing of influenza epidemic activity in China

Influenza season	2010/2011	2011/2012	2012/2013	2013/2014	2014/2015		2015/2016	2016/2017		2017/2018	2018/2019	2019/2020	2020/2021	2021/2022
Epidemic number	One	One	One	One	Two		One	Two		One	One	One		One
Starting time	52	1	50	49	50	24	53	48	26	48	51	47	/	49
Ending time	12	14	19	14	14	32	18	12	35	12	18	7	/	11
